# Dietary guidance for pregnant women using DeepSeek-R1 and ChatGPT-4.0: a comparative analysis

**DOI:** 10.3389/fpubh.2026.1728954

**Published:** 2026-02-03

**Authors:** ZeJun Gao, Jie Li, WeiYue Fang

**Affiliations:** 1 Department of Hematopathology, The Second Affiliated Hospital and Yuying Children’s Hospital of Wenzhou Medical University, Wenzhou, Zhejiang, China; 2 Department of Pediatric, The Second Affiliated Hospital and Yuying Children's Hospital of Wenzhou Medical University, Wenzhou, Zhejiang, China

**Keywords:** artificial intelligence, ChatGPT, DeepSeek, diet, DQI-I, nutrition, pregnancy

## Abstract

**Background:**

Advancements in artificial intelligence (AI) and natural language processing have enabled the widespread application of large language models. However, the ability of AI models to provide dietary guidance for pregnant women remains unclear. This study aims to explore the capabilities of DeepSeek-R1 and ChatGPT-4.0 in generating dietary plans for pregnant women with different activity levels.

**Methods:**

Personalized diet plans were generated using DeepSeek-R1 and ChatGPT-4.0. Through calorie calculation, Diet Quality Index-International (DQI-I) assessment, and cost analysis, the dietary quality and cost performance were evaluated.

**Results:**

The requested caloric targets in DeepSeek’s diet plans were superior to those of ChatGPT. All plans achieved a satisfactory DQI-I score (≥ 70). The “adequacy” score of DeepSeek-R1 was much higher (DeepSeek-R1 35.8 ± 0.7 vs. ChatGPT-4.0 33.9 ± 0.8, *p* < 0.001), while ChatGPT-4.0 performed better in the “moderation” aspect (ChatGPT-4.0 22.3 ± 2.2 vs. DeepSeek-R1 17.0 ± 3.4, *p* = 0.004). ChatGPT-4.0 demonstrated better performance in terms of cost-effectiveness (*p* = 0.017).

**Conclusion:**

This study shows that DeepSeek-R1 and ChatGPT-4.0 can be helpful in providing personalized and reasonable dietary advice for pregnant women. In some aspects, such as food type adequacy, the emerging model “DeepSeek” performs better than ChatGPT.

## 1 Introduction

The importance of nutrition during pregnancy in relation to pregnancy outcomes has long been acknowledged. When the intake of nutrients does not match the nutritional requirements, it may lead to adverse health effects (1, 2). Malnutrition is considerably prevalent in countries and regions with relatively underdeveloped economies (3). Insufficient iron intake can lead to iron deficiency anemia (4). Calcium intake mainly impacts the growth and development of bones (5). In addition to preventing scurvy, previous studies have found vitamin C is associated with chronic heart disease, postmenopausal breast cancer, stroke, etc. (6). Apart from its significance in pregnant women, good maternal nutritional status is also critical for the development of the fetal brain and nervous system (7, 8). Furthermore, with the development of the world economy and technology, obesity is increasingly becoming a serious epidemic and is drawing much public attention. It is now so common that it is replacing the more traditional public healthcare concerns, including malnutrition and infectious diseases, as one of the most significant contributors to ill health (9). Therefore, a healthy and reasonable diet is extremely important.

Currently, the core objective of prenatal nutrition guidance has shifted from merely meeting basic nutrient requirements to preventing adverse maternal and infant outcomes by optimizing the overall dietary pattern. The latest systematic review by the Advisory Committee of the 2025 Dietary Guidelines of the United States indicates that a diet pattern emphasizing the intake of vegetables, fruits, legumes, nuts, whole grains, fish, and dairy and lower intakes of added sugars may be associated with a reduced risk of excessive gestational weight gain, although the current evidence level is still rated as ‘limited’ (10). This indicates that personalized dietary guidance remains a challenge. In the past, people turned to dietitians for professional and reliable assistance (11). However, there was another group of women who often developed incorrect notions about prenatal nutrition due to misconceptions passed down by their parents or fallacies in popular traditions (12–14).

With advancements in large language models, these artificial intelligence (AI)-powered systems can generate personalized recommendations in various fields, including healthcare and nutrition (15). Currently, there are studies on AI-based dietary guidance for irritable bowel syndrome, cancer, and diabetes. The results show that these large language models can provide some assistance in dietary guidance (16–18). Other methods used ChatGPT to estimate the energy content and macronutrients of food based on images (19). However, research on dietary AI recommendations for pregnant women remains underexplored.

To assess the dietary nutritional status of residents and thereby reduce the occurrence of nutrition-relevant diseases, various comprehensive dietary quality assessment methods have been established worldwide (20). The Diet Quality Index (DQI), the first food-based priori index, was developed to measure diet quality that reflected the risk gradient for non-communicable chronic diseases (21). DQI-I, revised in 2003, focuses on four major aspects of a high-quality healthy diet: variety, adequacy, moderation, and overall balance (22).

By incorporating DQI-I and cost performance indicators, this study aims to explore the initial capabilities of two AI models in generating dietary plans for pregnant women during different stages of pregnancy. It is intended that this will help women address potential issues they may encounter in the future.

## 2 Materials and methods

### 2.1 Study design

This study employed a design to determine whether AI-driven chatbots can provide personalized and reasonable dietary advice for pregnant women, focusing on the initial responses of ChatGPT-4.0 and DeepSeek-R1. The AI pregnancy dietary project utilized two software programs, ChatGPT-4.0 and DeepSeek-R1, due to their “deep thinking” mechanism. To minimize the influence of previous user interactions, a new email account was created and used to log into each chatbot, ensuring that each AI’s responses were unaffected by prior learning. To ensure that each diet plan did not interfere with the others, each participant had set up a new chat window. The following content was entered into the dialog box: “Prepare a healthy daily meal plan for a 25-year-old woman in the early/s/third trimester of pregnancy, including portion sizes in grams, height 160 cm, weight 60 kg, and physical activity level: mild/moderate/high.” We focused on the initial response of the model; therefore, we only studied the first diet plan generated by the model. The question-and-answer session was conducted on 7 June 2025. A total of 18 AI diet plans were generated according to different pregnancy periods and physical activity levels (PALs).

The estimated energy requirement (EER) for adults was calculated using the Factorial Approach Method. According to the Food and Agriculture Organization of the United Nations, PAL is classified into three types, with coefficients ranging from low to high as 1.4, 1.7, and 2.0, respectively (23). Based on the Chinese Dietary Reference Intakes 2023 (CDRI 2023), extra energy requirements during the three pregnancy stages are as follows: 0, 250, and 400 kilocalories (24). The DQI-I was used to assess the rationality of the dietary plans. Four major categories, variety, adequacy, moderation, and balance, were evaluated successively (25). To ensure data accuracy and credibility, nutrient information, including energy for each food item, was verified and scored based on USDA FoodData Central and CDRI 2023 (24, 26). To eliminate bias in price fluctuations, we conducted a price search for relevant food items on the Tmall online platform with tourist status on the same day, 7 August 2025 (27).

### 2.2 Statistical analysis

SPSS 27.0 software (IBM Corp.) was used for statistical analysis, and WPS Office Excel was used for data visualization and graph plotting. The cost performance of the AI recipe is defined as the DQI-I score divided by the price. The mean and standard deviation were calculated for the DQI-I score, food price, and cost performance of each chatbot. The data were normalized using the Min–Max Normalization method and are presented in a radar chart. For extremely large (benefit indicator) attribute data, the normalization process is (X-XMIN)/(XMAX-XMIN), whereas for extremely small (cost indicator) attribute data, the normalization formula is (XMAX-X)/(XMAX-XMIN). The Shapiro–Wilk test was used to check whether the data were normally distributed, and Levene’s test was used for homogeneity of variance. According to normality and homogeneity of variance, Paired Samples t-test, Independent Samples *t*-Test, and Mann–Whitney U test were used for corresponding comparisons. Differences were considered statistically significant at a *p*-value of <0.05.

## 3 Results

### 3.1 Caloric comparison of diets

**Figure 1:**
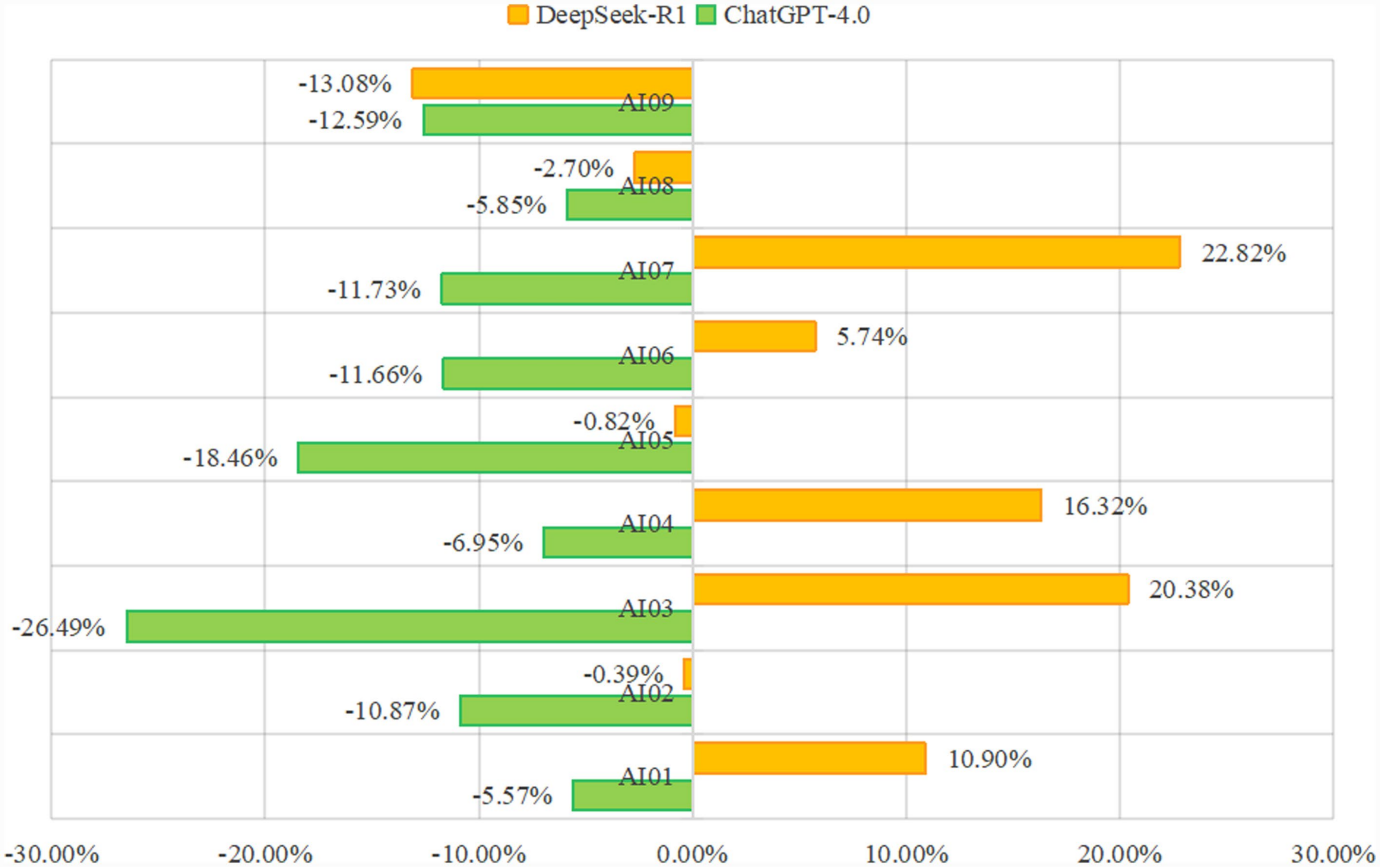
Energy percentage differences of DeepSeek and ChatGPT compared with estimated energy requirement.

The calorie intake target set by the DeepSeek diet plan was higher than that of ChatGPT (*p* = 0.007). The caloric values of the diets established by ChatGPT were generally lower than those of EER, whereas the calorie content of the diets produced by DeepSeek was relatively more sufficient. All diet plans generated by ChatGPT exceeded the requested calorie target by more than 5%, whereas in DeepSeek, the percentage within a 5% calorie difference accounted for 33% (3/9). (Figure 1). Table 1 presents the specific calorie values and proportions of macronutrients for each AI diet plan.

**Table 1:** AI diet plans’ calorie value and macronutrient content.

AI models	Meal plan	Pregnancy	PAL	EER (kcal)	Energy AI (kcal)	Protein (%)	Total Fat (%)	CHO (%)
ChatGPT-4.0	01	Early	Mild	1839.6	1737.1	26.45	33.69	42.36
	02	Early	Moderate	2233.8	1990.9	26.03	31.20	45.03
	03	Early	High	2628.0	1931.9	28.84	31.79	41.89
	04	Second	Mild	2089.6	1944.5	25.11	35.59	42.64
	05	Second	Moderate	2483.8	2025.4	26.55	33.51	41.78
	06	Second	High	2878.0	2542.6	24.20	37.60	40.82
	07	Third	Mild	2239.6	1977.0	26.20	34.35	42.20
	08	Third	Moderate	2633.8	2479.7	24.59	36.20	41.97
	09	Third	High	3028.0	2646.8	24.20	30.84	46.99
DeepSeek-R1	01	Early	Mild	1839.6	2040.2	22.17	36.20	45.77
	02	Early	Moderate	2233.8	2225.1	20.69	37.21	46.95
	03	Early	High	2628.0	3163.6	23.74	35.00	43.92
	04	Second	Mild	2089.6	2430.7	25.78	32.75	44.18
	05	Second	Moderate	2483.8	2463.6	24.59	35.09	43.90
	06	Second	High	2878.0	3043.2	24.03	38.19	41.30
	07	Third	Mild	2239.6	2750.6	24.14	36.67	42.90
	08	Third	Moderate	2633.8	2562.7	27.41	35.13	40.05
	09	Third	High	3028.0	2631.8	23.92	39.97	38.92

PAL, Physical activity level; EER, Estimated energy requirement.

### 3.2 Food difference

**Figure 2:**
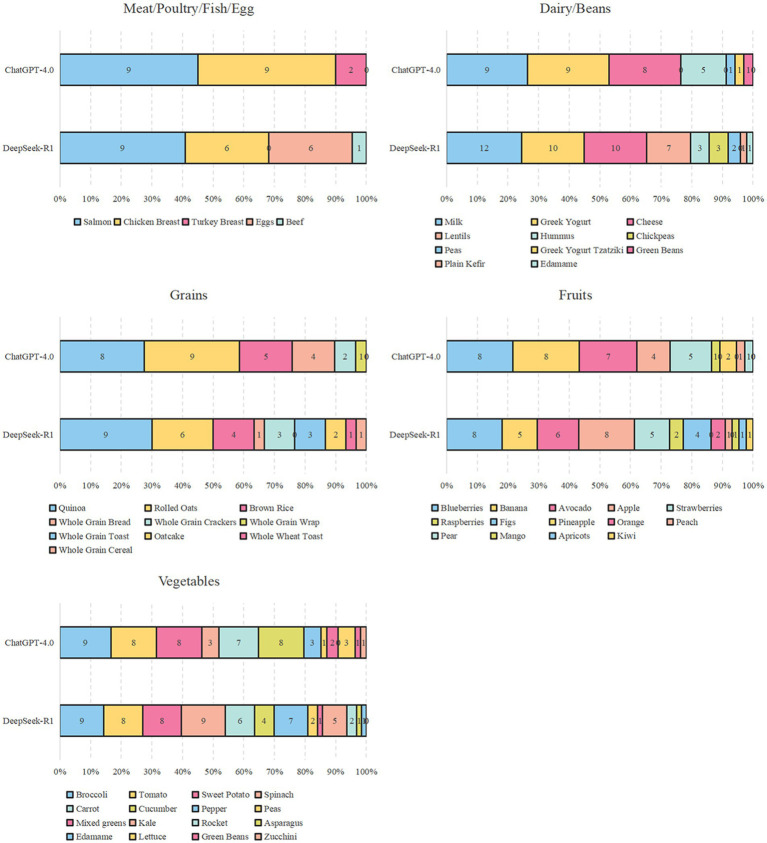
Specific items of “variety-food groups” of different diet plans generated by DeepSeek and ChatGPT.

**Figure 3:**
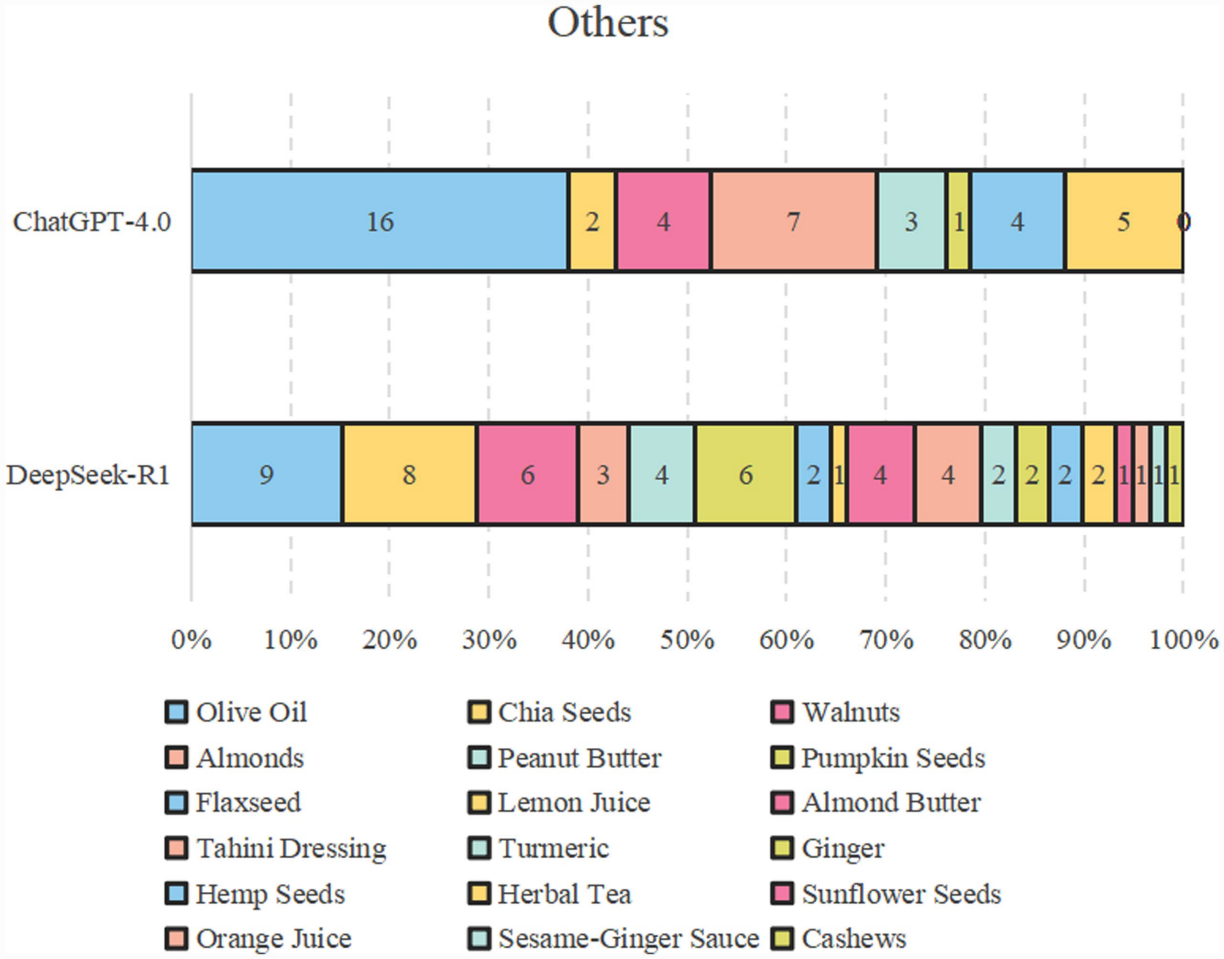
Unclassifiable food items of DeepSeek and ChatGPT.

**Figure 4:**
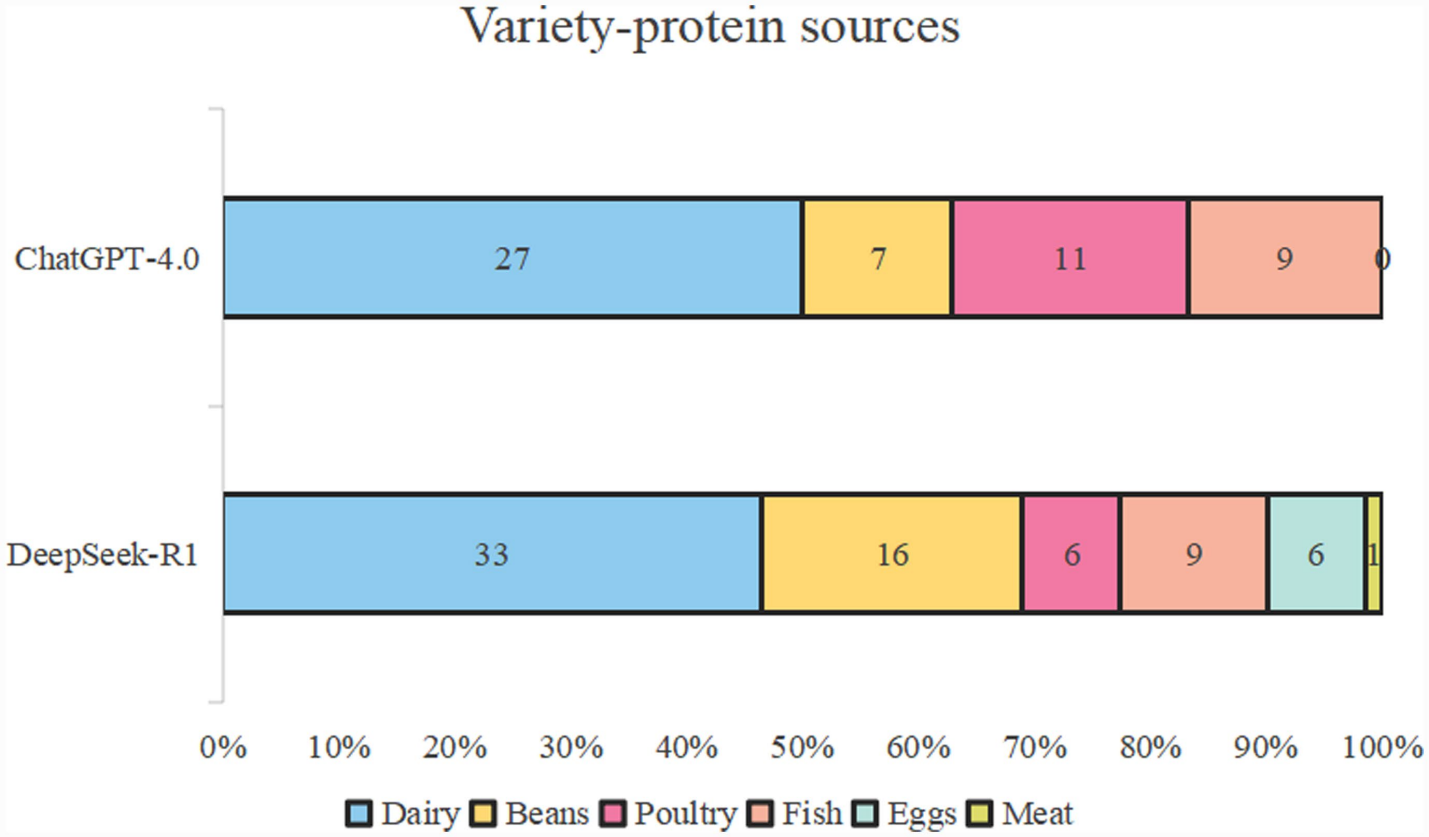
Items of the “variety-protein sources” section of DeepSeek and ChatGPT.

Figure 2 shows the specific items of the “variety” dietary categories of the two AI bots. For “meat/poultry/fish/egg” of the variety food groups, the first two items were both chicken breast and salmon. In all the AI diet plans, ChatGPT did not mention eggs, whereas DeepSeek mentioned eggs a total of six times, the same as the number of chicken breasts. For “dairy/beans,” both models highly recommended milk, cheese, and Greek yogurt. The difference is that ChatGPT recommended choosing either semi-skimmed or fully skimmed milk, whereas DeepSeek used full milk as the default option. As for “grains,” the former items were quinoa, rolled oats, and brown rice. Following these, there were different types of grain-based crackers or bread rolls. In terms of “fruits,” the first five items were blueberries, bananas, avocados, apples, and strawberries, accounting for over 70%. DeepSeek mentioned more types, such as figs, oranges, and mangoes. As for the category of “vegetables,” both mentioned broccoli, tomato, sweet potato, spinach, carrot, cucumber, and pepper. Figure 3 presents the other food types of ChatGPT and DeepSeek. It can be seen that ChatGPT was more enthusiastic about olive oil. DeepSeek, in addition to recommending the olive oil, also mentioned more seasonings and nuts, such as tahini dressing, turmeric, ginger, and a variety of seed foods. In terms of “variety-protein sources,” as shown in Figure 4, the first protein source recommended by both was dairy products, accounting for approximately 50%. ChatGPT suggests poultry and fish, while DeepSeek mentioned all six types of protein sources, preferring dairy and beans.

### 3.3 DQI-I score

Table 2 presents DQI-I scores of two AI model diet plans. DQI-I scores of ChatGPT’s diets were higher than DeepSeek’s; however, there was no statistical difference (ChatGPT-4.0 76.2 ± 2.4 vs. DeepSeek-R1 73.4 ± 3.1, *p* = 0.090). In “variety-food groups” and “variety-protein sources,” both had a maximum score. For “adequacy” and “moderation” aspects, DeepSeek-R1 performed better in the former (DeepSeek-R1 35.8 ± 0.7 vs. ChatGPT-4.0 33.9 ± 0.8, *p* < 0.001), while ChatGPT performed better in the latter (ChatGPT-4.0 22.3 ± 2.2 vs. DeepSeek-R1 17.0 ± 3.4, *p* = 0.004). As for “balance,” ChatGPT-4.0 achieved 0.0 ± 0.0, while DeepSeek-R1 achieved 1.1 ± 1.8 (*p* = 0.066).

**Table 2:** Mean and standard deviation of DQI-I scores and each category score for ChatGPT-4.0 and DeepSeek-R1.

AI models	Variety food groups	Variety protein sources	Adequacy	Moderation	Balance	DQI-I
ChatGPT-4.0	15.0 (0.0)	5.0 (0.0)	33.9 (0.8)	22.3 (2.2)	0.0 (0.0)	76.2 (2.4)
DeepSeek-R1	15.0 (0.0)	5.0 (0.0)	35.8 (0.7)	17.0 (3.4)	1.1 (1.8)	73.4 (3.1)
*p*-value	1	1	< 0.001	0.004	0.066	0.090

### 3.4 Cost performance

**Figure 5:**
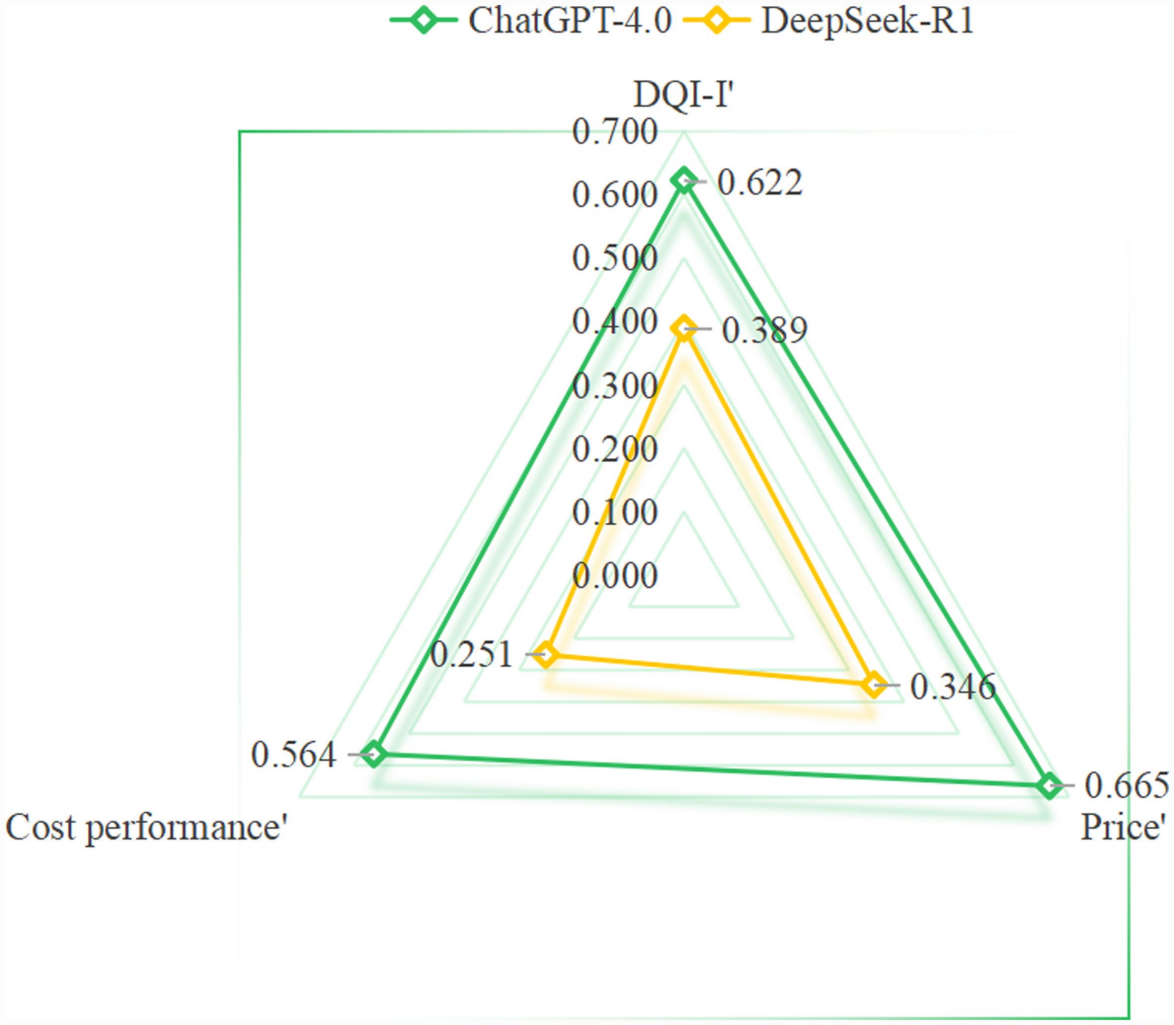
A radar chart is used for comparing the AI diet created by DeepSeek and ChatGPT (data are normalized).

As previously mentioned, ChatGPT’s diets had higher DQI-I scores. In terms of food price, the raw food materials of ChatGPT’s diets were cheaper than those of DeepSeek (ChatGPT-4.0 84.54 ± 11.60 vs. DeepSeek-R1 105.27 ± 19.36, *p* = 0.014), and it had a better cost-performance ratio (ChatGPT-4.0 0.917 ± 0.130 vs. DeepSeek-R1 0.729 ± 0.168, *p* = 0.017) (Table 3). The radar chart (Figure 5) illustrates that the diet plan of ChatGPT is superior overall.

**Table 3:** Comparison of the mean and standard deviation of the AI diet’s cost performance of ChatGPT-4.0 and DeepSeek-R1.

AI models	DQI-I	Price (CNY)	Cost performance
ChatGPT-4.0	76.2 (2.4)	84.54 (11.60)	0.917 (0.130)
DeepSeek-R1	73.4 (3.1)	105.27 (19.36)	0.729 (0.168)
*p*-value	0.090	0.014	0.017

## 4 Discussion

After years of development and practice, there are now indicators that are applicable to various situations to assess the nutritional status (28). DQI, proposed by Fred Hutchinson Cancer Research Center, is a comprehensive tool for evaluating the nutritional quality of dietary patterns (21). DQI-I covers the four main aspects of dietary patterns, namely diversity, adequacy, moderation, and balance, and is suitable as a comprehensive dietary quality assessment tool for different national populations (29). This study aims to explore the capabilities of AI models in generating dietary plans for pregnant women. It is hoped that this will help women cope with potential issues in the future.

Overall, in both models, the performance of the AI diets in terms of calorie control was not satisfactory. The energy value set by ChatGPT-4.0 for the diet was lower than EER, while the calorie content of the diet formulated by DeepSeek-R1 was higher than EER. The diet plan of DeepSeek-R1 performed relatively better in terms of energy accuracy. The proportion of DeepSeek diet plans with a calorie difference within 5% was 33% (3/9), while none of the dietary plans generated by ChatGPT-4.0 fell within this 5% range. Energy demands and physical activity increase as the trimester progresses (29). However, no significant trend of this type was observed in the two AI models. AI models may not be able to fully understand complex issues, such as “different trimesters combined with different PALs,” which leads to deviations in energy calculations. Due to the wide range of training sources and the inconsistent quality of the training data, there can sometimes be situations where inaccurate or fictional content, known as AI hallucination, is generated. Although these contents may seem reasonable, they are, in fact, inconsistent with the actual data (30, 31). Therefore, different calorie requirements were provided by the two models. For example, in the dietary plan formulated based on the early pregnancy stage and the high PAL, the EER was 2,628 kcal. ChatGPT-4.0 recommends an intake of 1931.9 kcal (−26.49%), while DeepSeek-R1 suggests 3163.6 kcal (20.38%). Both of the AI models showed a difference of more than 20% compared to EER. The calorie intake in ChatGPT’s diet is too low, while that in DeepSeek-R1’s diet is too high. To reduce or prevent the occurrence of inaccuracies, using repetitive questioning techniques may enhance the quality of responses, such as the three-turn iterative prompting approach previously proposed by others (32). However, since we are focusing on the model’s initial response to ensure and enhance its accuracy, improving the quality of the training data and providing simple and understandable prompts might be the most direct approach. In the future, we should explore ways to reduce these disparities, particularly to enhance the accuracy of the initial response.

In terms of DQI-I scores, both ChatGPT-4.0 and DeepSeek-R1 achieved good results (≥ 70). Compared to DeepSeek-R1, ChatGPT-4.0 achieved a higher DQI-I score. This may be attributed to the earlier history of invention, a longer development process, and a more established ecosystem (33). ChatGPT-4.0 and DeepSeek-R1 both achieved perfect scores in the dietary diversity (both food groups and protein sources) assessment. However, there are subtle differences in dietary recommendations, and DeepSeek-R1 is significantly more diverse in terms of food groups and protein sources, reducing diners’ boredom with a single food item. DeepSeek mentioned all six types of protein sources, namely dairy, beans, fish, poultry, eggs, and meat; however it is worth noting that the only type of meat mentioned was beef, which was referenced only once. The types of fish in these two models are limited to salmon, whereas the types of poultry are restricted to chicken and turkey, indicating that the variety of food options is still limited. This might be explained by the different regional developers of the model, reflecting the differences in the source of the background database and the differences between Oriental and Occidental cultures.

Balanced and reasonable dietary nutrition is essential for people’s growth and development as well as for a healthy lifestyle, and its significance for pregnant women is also self-evident. When the intake of nutrients does not match their demand, adverse health effects may occur (34, 35). In addition, excessive nutrition can have an impact on health, such as obesity (9). Thus, a healthy and balanced diet is extremely important. The “adequacy” module received good scores in both models, and the performance of DeepSeek-R1 was even more outstanding. The most obvious difference was in the iron content. Insufficient iron intake can lead to anemia, particularly during pregnancy, and is associated with serious outcomes such as preterm birth, low birth weight, and offspring neurodevelopmental disorders (36–38). In contrast, ChatGPT-4.0 scored higher in the “moderation” aspect. It places greater emphasis on the control of food nutrient content; i.e., it has imposed restrictions on foods that are not recommended to be consumed excessively (such as fat, sodium, and empty calorie foods), especially the strict control of cholesterol. For example, when it comes to the choice of dairy, ChatGPT usually opts for skimmed or semi-skimmed varieties, but DeepSeek does not pay any attention to this.

Regarding the “balance” module, the results of these two models were unsatisfactory. The poor macronutrient ratio resulted in none of the patients receiving any score. In particular, the energy proportion of total fat has always remained at a very high level, which is similar to previous studies (39). Optimal fatty acid distribution covering the balance of polyunsaturated, monounsaturated, and saturated fatty acids is also not satisfactory. In this evaluation, only three out of 18 diet plans received this “balance” score, and all of these scores were generated by DeepSeek-R1. In addition, it is also worth noting that, apart from olive oil, DeepSeek mentioned more seasonings, paying attention to the existence of taste buds, thereby enhancing the palatability of diners. Overall, these two models are simply “artificial idiots” when it comes to macronutrient balance and fatty acid distribution, which are closely related to health (40–42). Currently, AI models are still unable to precisely regulate the balance of various fatty acids and macronutrients. DeepSeek-R1 is slightly better in this regard. This is a significant limitation of current AI diets. Relying solely on these dietary methods without proper assessment may increase the health risks associated with the diet. Therefore, professional nutritional guidance is crucial for reducing these risks.

Nutrition-related diseases are related to the economic level of countries and regions. In those relatively underdeveloped economies, malnutrition is quite common, while excessive nutrition is more common in those developed regions (3). Cost-effective dietary guidance can be more beneficial for ordinary people. On cost performance, ChatGPT-4.0 demonstrated greater advantages. The diet plans of ChatGPT-4.0 can take into account the prices of food materials while achieving a similar DQI-I score, which is highly attractive for families or regions with limited budgets.

One major limitation of this study is the lack of clinical validation. Pregnant women can use these diet plans as a general reference, but if they were to follow them only, there might be certain ethical issues involved. This directly poses a safety risk and may lead to incorrect judgments regarding energy or key nutrient intake. If such data are adopted without verification, it could potentially harm the health of pregnant women and their fetuses. In the future, more in-depth and comprehensive clinical validation and evaluation will be of great significance. To better suit practical applications, based on the findings of this study, we propose a prospective clinical validation research framework consisting of an AI-generated diet plan and professional nutritionists’ dietary guidance. This will enhance the practicality and applicability of AI diet plans. Standardized input of core parameters (pregnancy stage, activity level), professional review of key contents (total calories, key nutrients, prohibited foods, etc.), adjustment of dietary cultural preferences, output, and continuous monitoring feedback will help healthcare professionals understand how to safely and effectively utilize AI-assisted nutrition planning. Although there are issues with variability in AI-generated outputs, these differences might require different daily dietary plans for choice. It will be necessary in the future to evaluate repetitive responses to the same background and assess the quality of each dietary plan. Second, since this study is a preliminary exploration of the ability of ChatGPT-4.0 and DeepSeek-R1 to generate prenatal diet plans, all the pregnant women in this study had no other chronic diseases, and their various dietary preferences were not taken into account. Indeed, it must be acknowledged that the diverse overall conditions of pregnant women in clinical settings make dietary guidance more complex. Therefore, it is necessary to conduct in-depth research to determine whether AI models can achieve good results in complex clinical situations. In addition, the number of diet plans in this study is limited. Incorporating specific scenarios of physical activities added into the study, such as doing 30 min of yoga every day and running 3 times a week, would contribute to meaningful research. Furthermore, to narrow the gap in food prices, we selected the initial prices of products from the same shopping website for unregistered accounts. However, we must admit that food prices vary seasonally and that there are also periodic fluctuations. Moreover, there are differences between different regions. In future research, it is necessary to compare food prices in different regions and consider the availability of local food, which will provide greater assistance to the general public.

## 5 Conclusion

This study shows that AI-driven chatbots, such as ChatGPT-4.0 and DeepSeek-R1, can be helpful in providing personalized and reasonable dietary advice for pregnant women, but they still cannot completely replace the expertise of trained professionals. During the entire pregnancy period, regardless of the PAL used, the calorie content of the diet plans generated by ChatGPT-4.0 was consistently lower and deviated more from the EER, whereas the calorie intake of DeepSeek-R1 was generally higher. The diet plan generated by DeepSeek-R1 will place more emphasis on flavor and offer a wider variety of food options. In contrast, ChatGPT-4.0 will be stricter in food control, especially in the strict control of cholesterol. ChatGPT-4.0 also performed better in terms of cost-effectiveness. In terms of macronutrient balance and fatty acid distribution, both performed poorly. If one solely relies on an AI-assisted diet, there may be issues such as unstable calorie intake and excessive and imbalanced intake of fatty acids. Supervision remains crucial for ensuring the reliability of the dietary recommendations generated by AI.

## Data Availability

The raw data supporting the conclusions of this article will be made available by the authors, without undue reservation.
